# Establishment and functional studies of a model of cardiomyopathy with cardiomyocyte-specific conditional knockout of *Arhgef18*

**DOI:** 10.1242/dmm.052172

**Published:** 2025-03-31

**Authors:** Xiaoqiong Fu, Wenjing Yuan, Jiajin Li, Kun Wan, Mei Ge, Bo Pan, Tiewei Lu

**Affiliations:** ^1^ Department of Cardiology, Children's Hospital of Chongqing Medical University, National Clinical Key Cardiovascular Specialty, National Clinical Research Center for Child Health and Disorders, Key Laboratory of Children's Important Organ Development and Diseases of Chongqing Municipal Health Commission, Ministry of Education Key Laboratory of Child Development and Disorders, China International Science and Technology Cooperation Base of Child Development and Critical Disorders, Chongqing Key Laboratory of Pediatrics, Chongqing Key Laboratory of Pediatrics, ChongQing 400014, China; ^2^ Chongqing General Hospital, Chongqing University, Chongqing 400014, China

**Keywords:** Cardiomyopathy, *Arhgef18*, Cytoskeleton, Cell polarity

## Abstract

The rising incidence of cardiomyopathies poses a significant threat to the physical and mental health of patients. The establishment of an animal model that accurately reflects the clinicopathological characteristics of cardiomyopathy is essential for investigating its pathogenesis. In this study, a cardiomyocyte-specific *Arhgef18* conditional knockout (cKO) mouse model was established with Cre/LoxP technology, and the results confirmed that the protein encoded by *Arhgef18* (Rho/Rac guanine nucleotide exchange factor 18) was knocked out effectively in the myocardium of *Arhgef18*^flox/flox^; *Nkx2.5-Cre* (*Arhgef18^fl/fl^* cKO) mice. Compared to *Arhgef18^fl/fl^* mice, *Arhgef18^fl/fl^* cKO mice presented with slower body weight growth and no differences in survival curves. Cardiac structure and function revealed that *Arhgef18^fl/fl^* cKO mice developed biventricular enlargement, ventricular wall thinning and left-ventricular systolic dysfunction, along with increased *Nppa* and *Nppb* mRNA expression levels. Additionally, *Arhgef18^fl/fl^* cKO mice showed cardiomyocyte cytoskeletal rearrangements and cell polarity disorders. Our study results suggest that *Arhgef18* cKO mice could provide an ideal animal model for the genetic investigation of cardiomyopathy.

## Teaser

**Summary:** We successfully established an *Arhgef18* conditional knockout mouse model, providing a new animal model for investigating the pathogenesis of cardiomyopathy.

## INTRODUCTION

Cardiomyopathies (CMs) are the most prevalent category of myocardial diseases that occur in childhood, and include dilated cardiomyopathy (DCM), hypertrophic cardiomyopathy (HCM) and left-ventricular noncompaction (LVNC). CMs are associated with severe clinical symptoms, poor prognoses and limited therapeutic options, and the aetiology remains largely unexplained (Maron et al., 2006; Mirza et al., 2022; Eldemire et al., 2024). Many studies have reported associations between CMs and variant genes of sarcomeres (Eldemire et al., 2020; Gao et al., 2024), ion channels (Shan et al., 2008; Mann et al., 2012) and mitochondria (Tang et al., 2010). However, these genetic factors account for only 20-30% of the CM phenotype. Notably, the same variants can result in diverse myocardiophenotypes. Furthermore, the phenotype remains unstable in previously reported genetic animal models (Shan et al., 2008; Mann et al., 2012; Wu et al., 2022; Rhee et al., 2018). Consequently, establishment of an animal model with a stable phenotype to explore the pathogenesis of CM is important for advancing the prevention and treatment of such disease.

Our research group recruited a representative family with LVNC, and conducted whole-exome sequencing on the peripheral blood of the proband and their relatives. Additionally, we performed transcriptome sequencing on induced pluripotent stem cell-derived cardiomyocytes of the proband. With the integrated analysis of transcriptome and whole-exome sequencing data, we identified the potential pathogenic gene Rho/Rac guanine nucleotide exchange factor 18 (*ARHGEF18*) (Li et al., 2022; Wan et al., 2023). *ARHGEF18* is a guanine nucleotide exchange factor that activates Rho GTPase by catalysing the conversion of GDP to GTP. It plays a crucial role in cytoskeletal rearrangement, cell proliferation and migration. Previous studies have reported the potential association of *ARHGEF18* with cardiovascular system development (Li et al., 2018). However, its involvement in the pathogenesis of CMs remains unclear. In this study, we used a gene-editing technology to construct a cardiomyocyte-specific *Arhgef18* conditional knockout (cKO) mouse model. Our aim was to investigate the functional role of *Arhgef18* in CMs by examining the associated CM phenotype. This work provides a reliable animal model for studying the genetic underpinnings of CMs.

## RESULTS

### Establishment and validation of the *Arhgef18* cKO mouse model

**Fig. 1.**
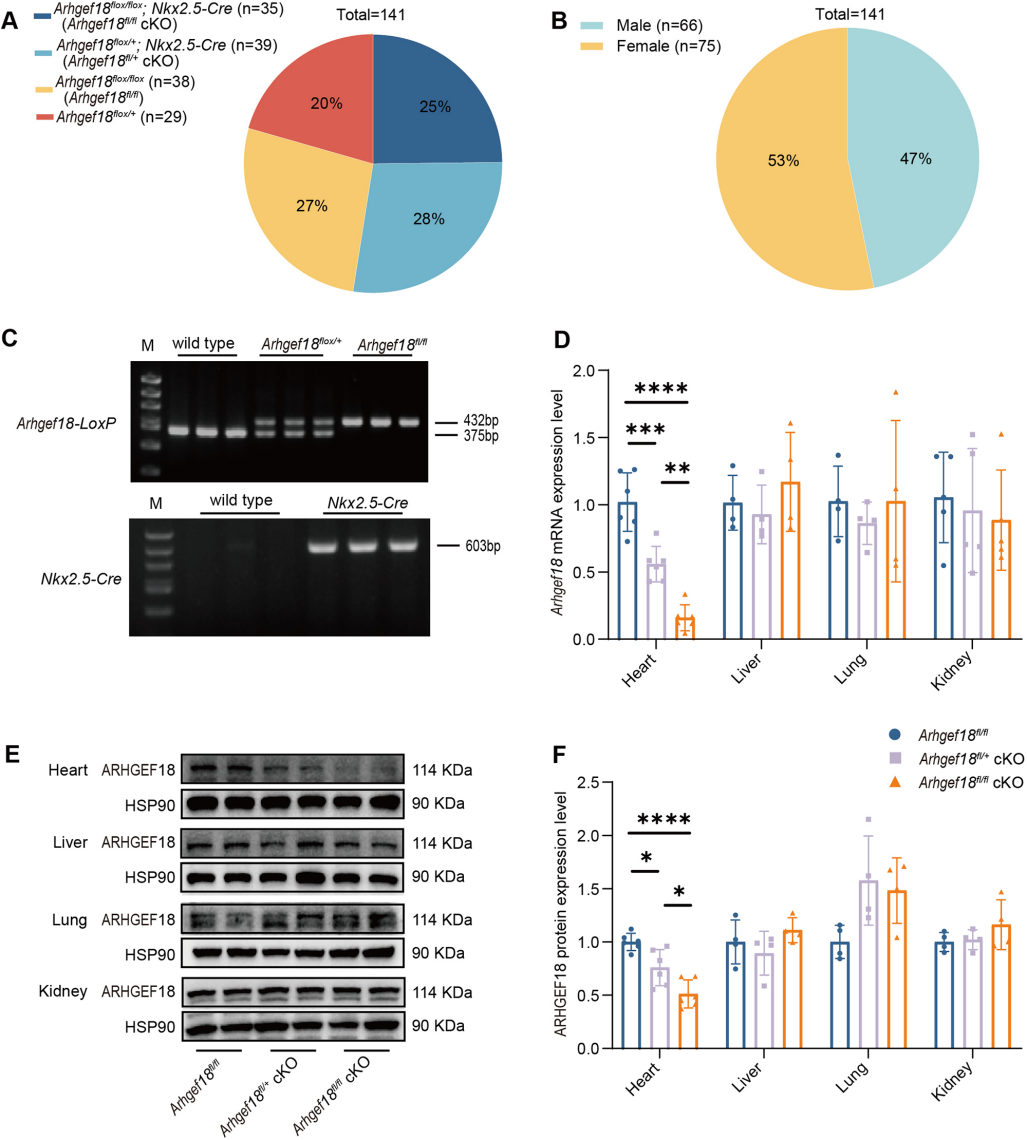
**The *Arhgef18* conditional knockout (cKO) mouse model was successfully constructed.** (A) Pie chart of four genotypes of F3 generation mice, approximately 1/4 each. (B) Pie chart of the genders of F3 generation mice, approximately 1/2 each. (C) Identification of mouse genotypes by PCR and agarose gel electrophoresis. Line M is a molecular size ladder for electrophoresis. The LoxP band was 432 bp, the wild-type band was 375 bp, and the Cre band was 603 bp in length. (D) *Arhgef18* mRNA expression in heart (*n*=6 per group), liver (*n*=4 per group), lung (*n*=4 per group) and kidney (*n*=5 per group) tissues from *Arhgef18^fl/fl^*, *Arhgef18^fl/+^* cKO and *Arhgef18^fl/fl^* cKO mice at 4 weeks of age by quantitative real-time PCR (q-PCR). (E,F) Expression levels of ARHGEF18 in heart (*n*=6 per group), liver (*n*=4 per group), lung (*n*=4 per group) and kidney (*n*=4 per group) tissues from three groups of mice at 4 weeks of age, detected using western blotting (E) and quantitatively analysed using HSP90 for normalization (F). The data are presented as the mean±s.d.. *P<0.05, **P<0.01, ***P<0.001, ****P<0.0001 versus respective control group (one-way ANOVA).

After two rounds of crosses between *Arhgef18^flox/+^* mice and *Nkx2.5-Cre* mice, a total of 141 offspring were obtained, including 35 *Arhgef18^flox/flox^; Nkx2.5-Cre* (*Arhgef18^fl/fl^* cKO) mice, 39 *Arhgef18^flox/+^; Nkx2.5-Cre* (*Arhgef18^fl/+^* cKO) mice, 38 *Arhgef18^flox/flox^* (*Arhgef18^fl/fl^*) mice and 29 *Arhgef18^flox/+^* mice, with each group accounting for approximately one-quarter of the total population (Fig. 1A). There were 53 female and 47 male mice, with each sex representing approximately half the sample size (Fig. 1B). These data suggest that *Arhgef18* cKO mice follow Mendelian inheritance patterns, and that mice with different genotypes are viable and fertilizable.

To investigate the potential role of *Arhgef18* in CMs, we selected *Arhgef18^fl/fl^*, *Arhgef18^fl/+^* cKO and *Arhgef18^fl/fl^* cKO mice for subsequent experimentation. PCR was employed to confirm the genotype (Fig. 1C). Mice with both 432 bp and 603 bp bands were classified as *Arhgef18^fl/fl^* cKO, whereas those with bands at 432 bp, 375 bp and 603 bp were classified as *Arhgef18^fl/+^* cKO. Subsequently, we assessed *Arhgef18* mRNA and protein expression levels in heart, liver, lung and kidney tissues at the age of 4 weeks. *Arhgef18* mRNA expression was decreased by ∼45%, and protein expression was decreased by ∼24%, in the heart of *Arhgef18^fl/+^* cKO mice compared to that of *Arhgef18^fl/fl^* mice. In addition, *Arhgef18* mRNA expression was decreased by ∼84%, and protein expression was decreased by ∼49%, in the heart of *Arhgef18^fl/fl^* cKO mice compared to that of *Arhgef18^fl/fl^* mice. Meanwhile, *Arhgef18* mRNA expression in the heart of *Arhgef18^fl/fl^* cKO mice was decreased by ∼72%, and protein expression was decreased by ∼33%, compared to that in the heart of *Arhgef18^fl/+^* cKO mice (Fig. 1D-F). Notably, there were no differences in *Arhgef18* expression between liver, lung or kidney tissues. These data suggested that *Arhgef18* was knocked down in the cardiomyocytes of *Arhgef18* cKO mice.

### *Arhgef18* cKO mice develop a CM phenotype

**Fig. 2.**
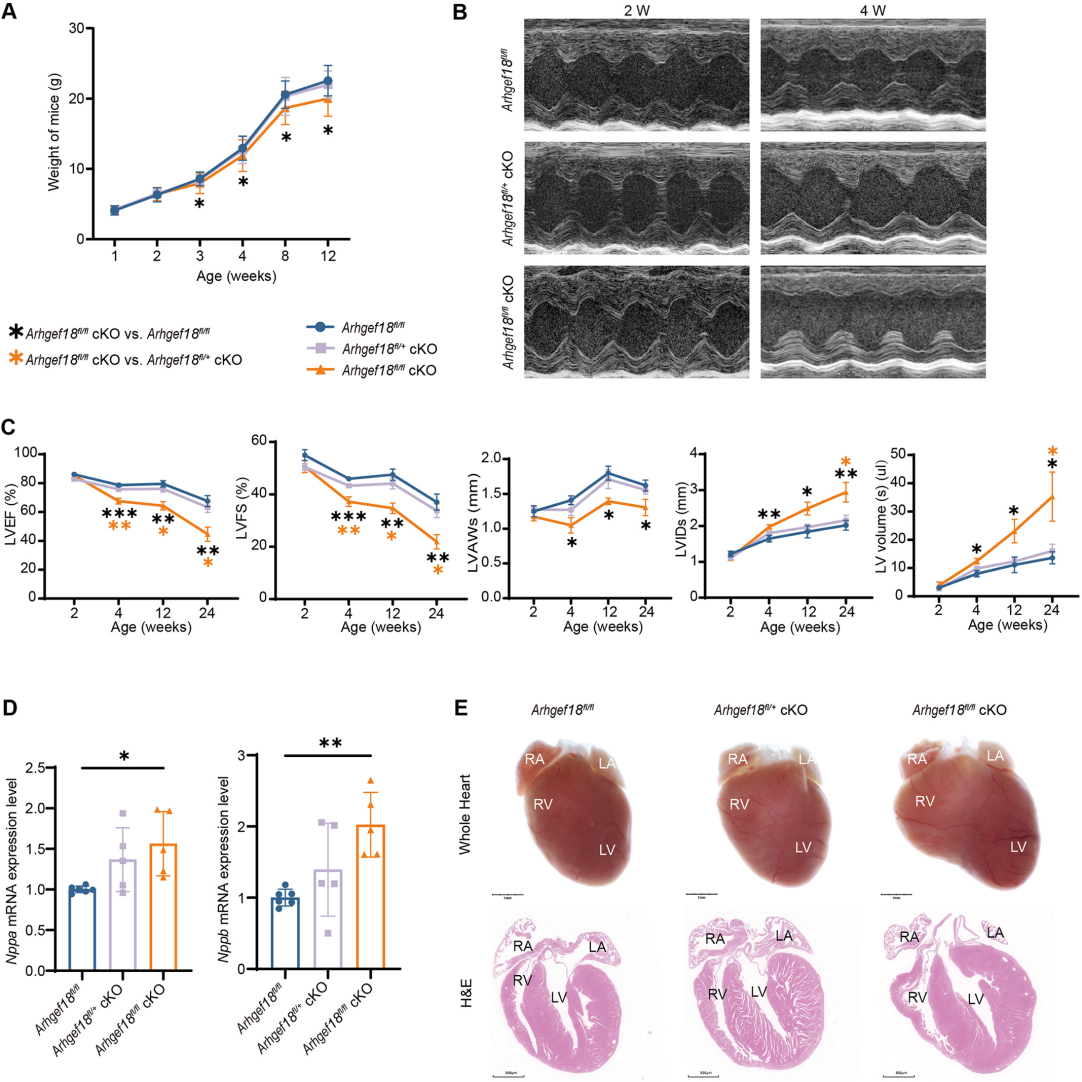
***Arhgef18* cKO mice develop a cardiomyopathy phenotype.** (A) Body weight of *Arhgef18^fl/fl^*, *Arhgef18^fl/+^* cKO and *Arhgef18^fl/fl^* cKO mice from the same litter at 1, 2, 3, 4, 8, and 12 weeks of age (*n*=40 per group for 1-4 weeks, *n*=20 per group for 8 weeks, *n*=15 per group for 12 weeks). *Arhgef18^fl/fl^* cKO mice presented pronounced deceleration of body weight growth beginning at 3 weeks of age. (B) Representative images of M-mode echocardiography of the heart in three groups of mice at 2 and 4 weeks of age. (C) Echocardiographic parameters of left-ventricular (LV) ejection fraction (LVEF), LV fraction shortening (LVFS), LV anterior wall thickness at end systole (LVAWs), LV inner diameter at end systole (LVIDs) and LV volume at end systole [LV volume (s)] were analysed in three groups of mice (*n*=5-8 mice per time point in each group) at 2, 4, 12 and 24 weeks of age, suggesting reduced systolic function, cardiac chamber enlargement and ventricular wall thinning in *Arhgef18^fl/fl^* cKO mice. (D) *Nppa* and *Nppb* mRNA expression in three groups of mice (*n*=5-6 per group) at 4 weeks of age by q-PCR, suggesting reduced cardiac function in *Arhgef18^fl/fl^* cKO mice. (E) Representative images of gross cardiac morphology (scale bars: 1 mm) and Haematoxylin and Eosin (H&E) staining (scale bars: 500 μm) of four-chamber heart sections from three groups of mice (*n*=3 per group) at 4 weeks of age, all showing biventricular enlargement in *Arhgef18^fl/fl^* cKO mice. LA, left atrium; LV, left ventricle; RA, right atrium; RV, right ventricle. The data are presented as the mean±s.d. *P<0.05, **P<0.01, ***P<0.001 versus respective control group (one-way ANOVA).

The body weight of the mice was measured at various time points during the cardiac development period. The results revealed no significant differences in body weight changes between *Arhgef18^fl/+^* cKO mice and *Arhgef18^fl/fl^* mice. However, compared with that of *Arhgef18^fl/fl^* mice, the growth weight of *Arhgef18^fl/fl^* cKO mice was slowed down after the age of 3 weeks (Fig. 2A). Additionally, the survival curves revealed no significant differences among the groups (Fig. S1A), indicating a potential growth restriction associated with alterations in cardiac morphology and function that does not impact survival. Consequently, cardiac systolic function was monitored over an extended period. Short-axis M-mode ultrasound images revealed that there were no significant differences in cardiac function or chamber size in *Arhgef18^fl/+^* cKO mice compared to *Arhgef18^fl/fl^* mice at any time point (Fig. 2B,C). Conversely, the cardiac function of *Arhgef18^fl/fl^* cKO mice declined progressively after 4 weeks, as evidenced by a reduction in the left-ventricular ejection fraction (LVEF) and left-ventricular fraction shortening (LVFS), accompanied by an increase in the left-ventricular inner diameter (LVID) and left-ventricular volume, as well as thinning of the left-ventricular anterior wall (LVAW) (Fig. 2B,C). These differences became increasingly pronounced with advancing age. Notably, similar differences were also observed between *Arhgef18^fl/+^* cKO and *Arhgef18^fl/fl^* cKO mice, with the data presented in Table S1. We also found that *Nppa* and *Nppb* mRNA expression levels within the ventricular muscle tissue of *Arhgef18^fl/fl^* cKO mice were significantly elevated compared with those within the ventricular muscle tissue of *Arhgef18^fl/fl^* mice (Fig. 2D). In summary, these findings indicate that *Arhgef18^fl/fl^* cKO mice exhibited early cardiac dysfunction at 4 weeks of age, followed by a progressive decline in cardiac function. However, overall survival rates were not negatively impacted in these mice.

The development of CM is a chronic and progressive process (Maron et al., 2006), thus necessitating the study of the early pathogenesis. In this study, we focused on the initial stages of CM phenotype development by examining 4-week-old mice. The gross examination and Haematoxylin and Eosin (H&E) staining of the whole heart revealed that *Arhgef18^fl/fl^* cKO mice exhibited significantly larger bilateral ventricular volumes than those of *Arhgef18^fl/+^* cKO and *Arhgef18^fl/fl^* mice (Fig. 2E). These findings indicate that *Arhgef18* cKO mice present a CM phenotype characterized by biventricular enlargement, thinning of the ventricular walls and diminished left-heart systolic function, confirming the successful establishment of a genetic animal model for CMs.

### Skeletal reorganization of myocardium in *Arhgef18* cKO mice

**Fig. 3.**
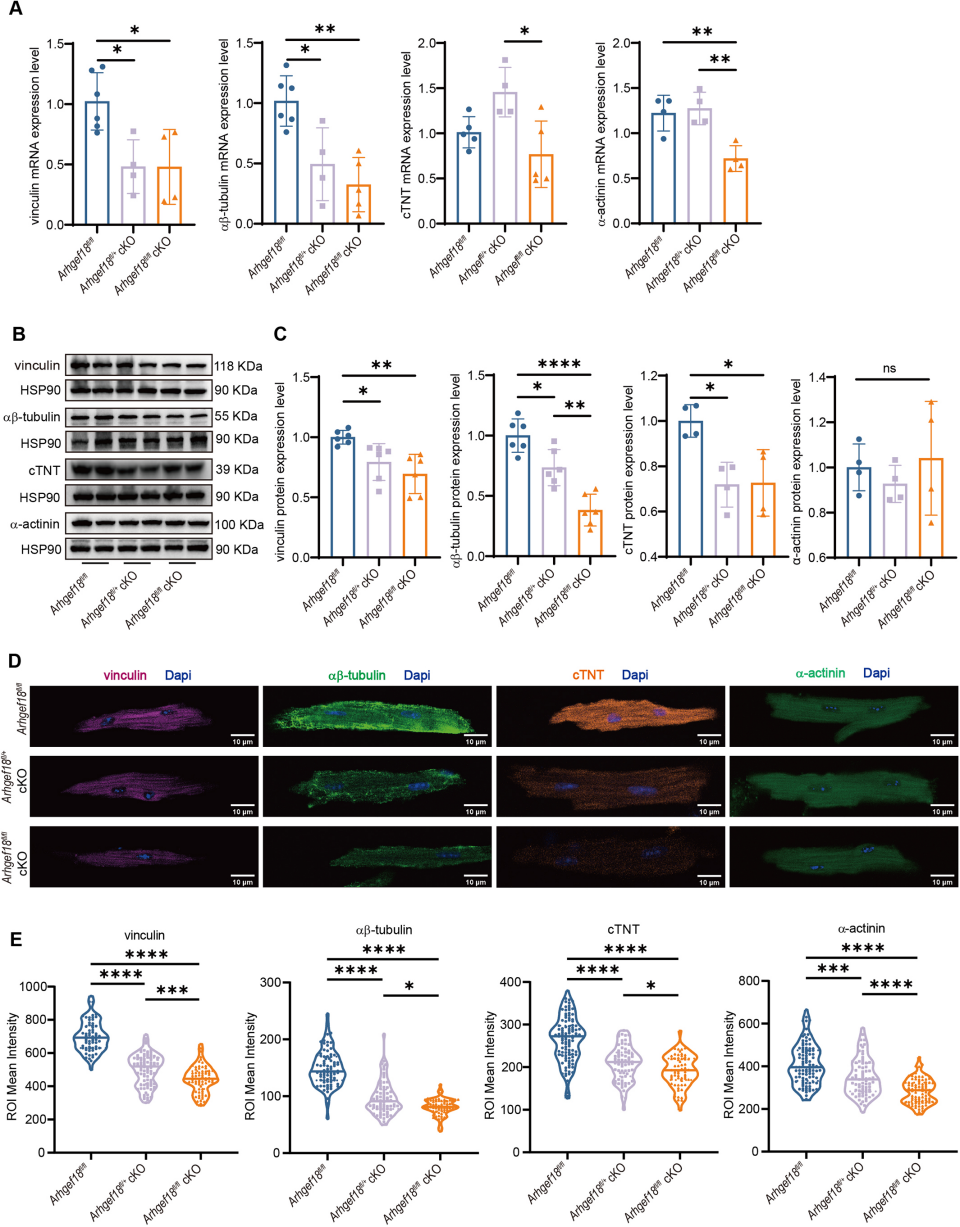
**Cytoskeletal rearrangement of myocardium in *Arhgef18* cKO mice.** (A) mRNA expression of cytoskeleton-related genes, including vinculin, αβ-tubulin, *cTNT* and α-actinin, in myocardial tissue from three groups of mice (*n*=4-6 per group) at 4 weeks of age by q-PCR, showing reduced expression in *Arhgef18* cKO mice. (B,C) The expression levels of cytoskeletal proteins, including vinculin, αβ-tubulin, cTNT and α-actinin, in myocardial tissue from three groups of mice (*n*=4-6 per group) at 4 weeks of age, detected using western blotting (B) and quantitatively analysed using HSP90 for normalization (C), suggesting that the expression of these proteins is lower in *Arhgef18* cKO mice than in *Arhgef18^fl/fl^* mice. (D,E) Fluorescence intensity of cytoskeletal proteins (E), including vinculin, αβ-tubulin, cTNT and α-actinin, in cardiomyocytes from three groups of mice (*n*=3 mice per group; >20 cardiomyocytes per mouse) at 4 weeks of age, detected using immunofluorescence (D), showing that the fluorescence intensity of these proteins in *Arhgef18* cKO mice was weaker than that in *Arhgef18^fl/fl^* mice. Scale bars: 10 μm. Blue, DAPI; purple, vinculin; dark green, αβ-tubulin; orange, cTNT; light green, α-actinin. ROI, region of interest. The data are presented as the mean±s.d. *P<0.05, **P<0.01, ***P<0.001, ****P<0.0001 versus respective control group (one-way ANOVA). ns, not significant.

*Arhgef18* is involved in cytoskeletal rearrangement (Zaritsky et al., 2017). Here, we aimed to explore the alteration of the cytoskeleton in *Arhgef18* cKO mice. We identified alterations in cytoskeletal proteins [vinculin, αβ-tubulin, cTNT (also known as TNNT2) and α-actinin] in the ventricles of 4-week-old mice with CM by quantitative real-time PCR (q-PCR) analysis of the encoding mRNAs and western blotting. In *Arhgef18^fl/fl^* cKO mice, the mRNA expression levels of cytoskeletal proteins such as vinculin, αβ-tubulin and α-actinin were significantly reduced compared to those in *Arhgef18^fl/fl^* mice (Fig. 3A). Similarly, the protein levels of vinculin, αβ-tubulin and cTNT were decreased compared to those in *Arhgef18^fl/fl^* mice (Fig. 3B,C). In *Arhgef18^fl/+^* cKO mice, mRNA levels of vinculin and αβ-tubulin were lower than those in *Arhgef18^fl/fl^* mice, with corresponding reductions in protein levels of vinculin, αβ-tubulin and cTNT (Fig. 3A-C). These findings suggest that *Arhgef18* plays a crucial role in maintaining cytoskeletal integrity in cardiomyocytes, and its knockout leads to downregulation of key cytoskeletal components, potentially contributing to observed cardiac dysfunction and CM phenotype.

Given the diverse cell types in myocardium, including cardiomyocytes, fibroblasts, smooth muscle cells and endothelial cells, we employed immunofluorescence to directly assess the impact of *Arhgef18* on the cytoskeleton of cardiomyocytes. Compared to *Arhgef18^fl/fl^* mice, *Arhgef18^fl/+^* cKO and *Arhgef18^fl/fl^* cKO mice exhibited reduced fluorescence intensities for cytoskeletal proteins, including vinculin, αβ-tubulin, α-actinin and cTNT, with the most pronounced decreases observed in *Arhgef18^fl/fl^* cKO mice (Fig. 3D,E). These data indicate that *Arhgef18* knockout results in reduced expression of cytoskeletal proteins in cardiomyocytes, suggesting the presence of cardiomyocyte skeleton rearrangements in *Arhgef18^fl/fl^* cKO mice.

### Disruption of the polarity of myocardial tissue in *Arhgef18* cKO mice

**Fig. 4.**
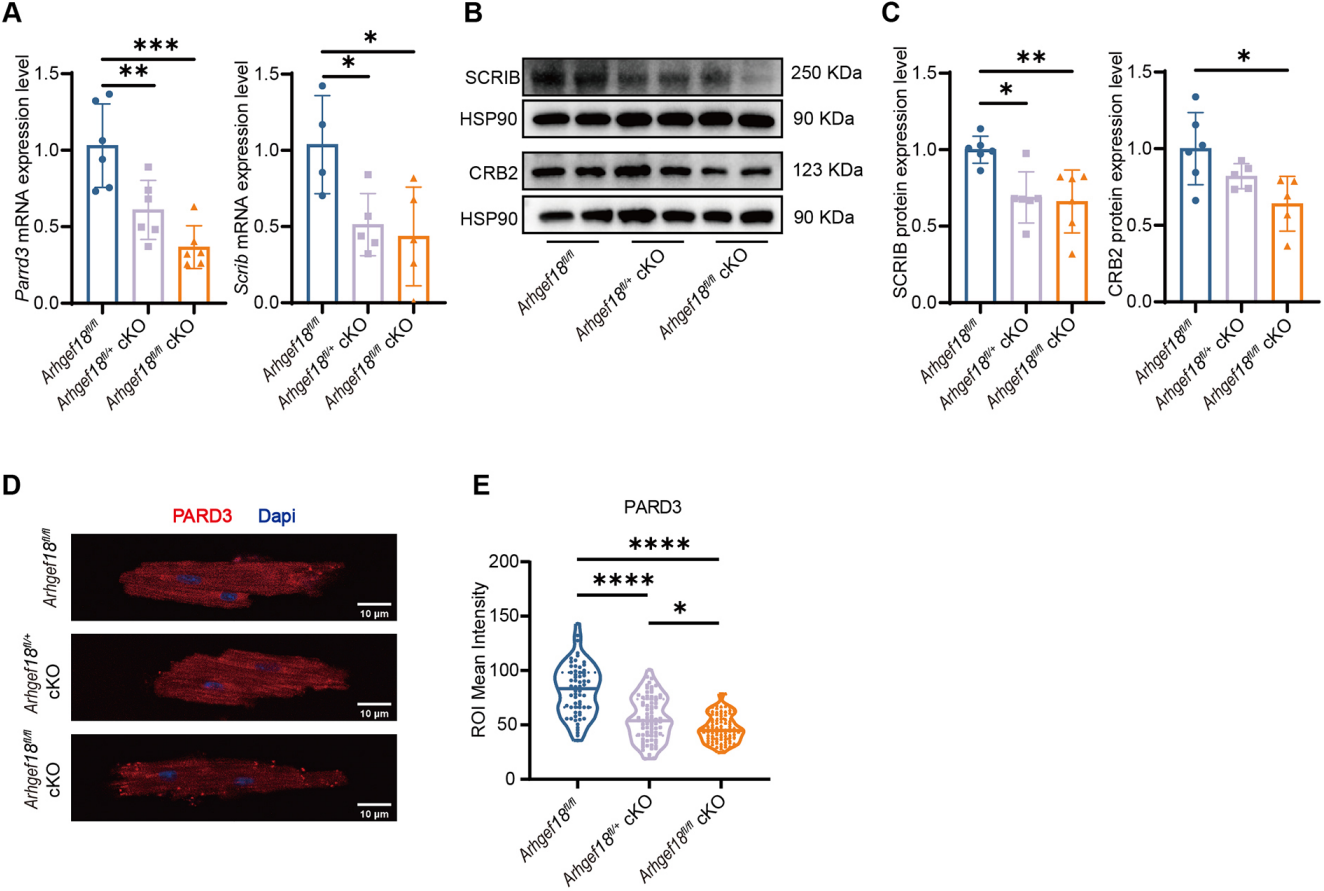
**Disruption of myocardial tissue polarity in *Arhgef18* cKO mice.** (A) The mRNA expression of polarity-related genes, including *Pard3* and *Scrib* in the myocardial tissue from three groups of mice (*n*=4-6 per group) at 4 weeks of age by q-PCR, showing reduced mRNA expression of polarity-related genes in *Arhgef18* cKO mice. (B,C) The expression levels of polarity proteins, including SCRIB and CRB2, in myocardial tissue from three groups of mice (*n*=5-6 per group) at 4 weeks of age, detected using western blotting (B) and quantitatively analysed using HSP90 for normalization (C), suggesting reduced expression of polarity proteins in *Arhgef18* cKO mice. (D,E) Fluorescence intensity of the polarity protein PARD3 (E) in cardiomyocytes from three groups of mice (*n*=3 mice per group; >20 cardiomyocytes per mouse) at 4 weeks of age, detected using immunofluorescence (D), showing that the fluorescence intensity of PARD3 in *Arhgef18* cKO mice was weaker than that in *Arhgef18^fl/fl^* mice. Scale bars: 10 μm. Blue, DAPI; red, PARD3. The data are presented as the mean±s.d. *P<0.05, **P<0.01, ***P<0.001, ****P<0.0001 versus respective control group (one-way ANOVA).

*Arhgef18* is also involved in regulation of cell polarity (Zaritsky et al., 2017; Herder et al., 2013). The expression level of cell polarity proteins (PARD3, SCRIB and CRB2) was validated via q-PCR analysis of the encoding mRNAs, western blotting and immunofluorescence. Compared to *Arhgef18*^fl/fl^ mice, both *Arhgef18^fl/fl^* cKO mice and *Arhgef18^fl/+^* cKO mice presented significantly lower *Pard3* and *Scrib* mRNA expression, and significantly lower SCRIB and CRB2 protein expression (Fig. 4A-C). Immunofluorescence further demonstrated a marked decrease in PARD3 protein localization and intensity in the *Arhgef18^fl/fl^* cKO mice compared to *Arhgef18^fl/fl^* mice (Fig. 4D,E). These data indicate that *Arhgef18* knockdown resulted in decreased expression of cardiomyocyte polarity proteins, suggesting the presence of myocardial tissue polarity disorders in *Arhgef18* cKO mice.

## DISCUSSION

In the present study, we successfully employed the Cre/LoxP system to construct an *Arhgef18* cKO mouse model. These mice exhibited CM phenotype characterized by bilateral ventricular enlargement, thinning of the ventricular walls and diminished cardiac contractile function. Additionally, the cytoskeleton rearrangements and cell polarity disorders were observed in *Arhgef18* cKO mice. These results suggest that *Arhgef18* is a pathogenic gene involved in the development of CMs and is potentially linked to the cytoskeleton and cell polarity.

The protein encoded by *ARHGEF18* is a guanine nucleotide exchange factor belonging to the Rho GTPase GEF family. *ARHGEF18* catalyses the conversion of GDP to GTP and directly activates Rho GTPases, which play critical roles in cytoskeletal rearrangement, cell growth and migration (Xu et al., 2019; Zaritsky et al., 2017; Mitchell et al., 2011). Most studies have indicated that the *ARHGEF18* plays a crucial role in regulating the polarity state of neuroepithelial cells and promoting retinal development by activating the RHOA–ROCK2 signalling pathway (Herder et al., 2013; Loosli, 2013; Arno et al., 2017). Clinical studies have reported that mutations in *ARHGEF18* lead to increased susceptibility to nonidiopathic pulmonary hypertension associated with coronary artery disease (Li et al., 2018), and systematic knockdown of *Arhgef18* results in embryonic death in mice (Beal et al., 2021). However, foundational research on *ARHGEF18* in the field of CMs has not been reported. Previously, a typical LVNC line was obtained from clinical samples and subjected to whole-exome sequencing in our group (Guo et al., 2021). Concurrently, transcriptome sequencing was performed on human induced pluripotent stem cell-derived cardiomyocytes, and Gene Ontology (GO) and Kyoto Encyclopedia of Genes and Genomes (KEGG) analyses of the transcriptome sequencing results revealed significant enrichment of many differentially expressed genes related to the cytoskeleton and cell polarity, and associated with the Rho GTPase cycle pathway (Guo et al., 2021). Ultimately, combining the results on whole-exome sequencing and transcriptome sequencing indicated downregulated expression of the pathogenic gene, *ARHGEF18* (Li et al., 2022; Wan et al., 2023). Phenotypic alterations caused by genetic abnormalities are the gold standard for validating the genetic basis of clinical disease, so the establishment and phenotypic validation of the *Arhgef18* cKO animal model could facilitate investigation of the mechanism of CMs. Our previous study used *Myh6-Cre* to construct an *Arhgef18* cKO mouse model that exhibits abnormal expression of cytoskeletal and polarity proteins in the myocardium but does not show CM-related phenotypes (Wan et al., 2023). The lack of phenotype was attributed to the late onset of recombination induced by *Myh6-Cre* [embryonic day (E)8.5]. To investigate the role and mechanism of *Arhgef18* in CM, we reconstructed the *Arhgef18* cKO mouse model using *Nkx2.5-Cre*, which initiates Cre recombinase activity earlier at E7.5.

In the present study, we observed that *Arhgef18^fl/fl^* cKO mice presented a phenotype characterized by bilateral ventricular enlargement, thinning of the ventricular walls and left-ventricular hypoconstriction beginning at 4 weeks of age. This finding is consistent with the pronounced deceleration in body weight growth observed in *Arhgef18^fl/fl^* cKO mice beginning at 3 weeks of age. Interestingly, the phenotype changed dynamically with the age of the *Arhgef18* cKO mice, and significant differences were observed between the *Arhgef18^fl/fl^* cKO and *Arhgef18^fl/+^* cKO mice. These phenotypic characteristics are consistent with the clinical presentations of DCM, with ∼30-40% of DCM aetiology being genetically linked (Khan et al., 2022). Numerous studies have indicated that common pathogenic genes associated with DCM – such as *TTN* (Vissing et al., 2021), *LMNA* (Le Dour et al., 2022), *MYH7* (de Frutos et al., 2022), *FLNC* (Verdonschot et al., 2020), *TNNT2* (Luedde et al., 2010) and *ZASP*/*Cypher* (also known as *LBD3*) (Lv et al., 2021; Vatta et al., 2003; Zheng et al., 2009) – predominantly encode cytoskeletal proteins, nuclear membrane proteins and ion channel proteins. Extensive research has established a correlation between DCM and LVNC. For example, LVNC has been identified in young carriers of lamin A/C mutations in familial cases of DCM (Hermida-Prieto et al., 2004). Other investigations have demonstrated that genetic mouse models carrying mutant genes identified in patients with LVNC, such as *TNNT2* (Luedde et al., 2010) and *ZASP*/*Cypher* (Vatta et al., 2003; Zheng et al., 2009), present typical morphological characteristics associated with DCM but lack the clinical phenotype characteristic of LVNC, and these findings are consistent with the results of our study. Our findings suggest that although numerous genes have been implicated in CMs to date, the intricate genetic mechanisms of CMs require further study, and new related genes could be discovered.

The pathogenic gene in this study, *ARHGEF18*, was identified within the LVNC family line. However, the *Arhgef18* cKO mice did not exhibit the LVNC-characteristic phenotype for several potential reasons. (1) The absence of *Yap1* in the dense layer of the myocardium leads to a thinner dense layer and trabecular overgrowth. Conversely, the lack of *Yap1* in the trabecular layer of the myocardium does not influence the growth of the dense layer (Tian et al., 2017). In this research, *Nkx2.5* (also known as *Nkx2-5*) – a marker for the trabecular myocardium – through which deletion of *Arhgef18* in mouse trabecular myocardium does not lead to thinning of the dense layer. This finding is consistent with those of previous studies (Tian et al., 2017) and reinforces that growth of the dense layer is largely independent of the trabecular layer. (2) Both *Myh6-Cre* (Huang et al., 2021) and *Nkx2.5-Cre* (Stanley et al., 2002) are cardiomyocyte-specific tools designed to knock out *Arhgef18* exclusively in cardiomyocytes. However, trabeculae formation and compaction involve multiple signal pathways originating from endocardial, myocardial and epicardial sources (Liu et al., 2018). Previous reports utilizing various Cre lines (*Myh6-Cre*, *Tie2-Cre*, *Nfatc1-Cre* and *Apj-CreER*) to downregulate *Ino80* expression in cardiomyocytes, epicardium or endothelial cells showed that only endothelial deletion during embryonic development results in an LVNC phenotype, with other cardiac cell types with *Ino80* knockdown not showing an associated phenotype (Rhee et al., 2018). Consequently, we propose developing a new *Arhgef18* cKO mouse model using endothelial-specific Cre mice or simultaneous cardiomyocyte- and endothelial-specific Cre mice. This approach offers promising avenues for investigating CMs caused by *Arhgef18*. (3) Environmental factors play an important role in the development of disease caused by genetic variants (Hazebroek et al., 2015; Thomas, 2004), and studies have reported that young *Prdm16* cKO mice exhibit normal cardiac function, but metabolic stress interventions induce heart failure and cardiac hypertrophy (Cibi et al., 2020). Therefore, the incorporation of stressors, including aortic arch narrowing surgery and high-fat chow feeding, provides avenues for validation of the phenotype of *Arhgef18* cKO mice. (4) Deletion of *ARHGEF18* appears to cause the LVNC phenotype in humans, but not in mice, suggesting a possible requirement for a ‘specific’ genetic background. This specific genetic background has also been reported in HCM, including large variation in cardiac hypertrophy among different mouse strains and even the absence of cardiac hypertrophy in some mice (Semsarian et al., 2001). Therefore, the phenotype of the mouse model is regulated by multiple factors, and phenotypic verification of the pathogenic gene could elucidate the genetic mechanism of disease onset. The inconsistent phenotype of the *Arhgef18* cKO mice in this study provides new ideas and research directions for exploring the genetics of CMs.

The cytoskeleton and cell polarity are crucial for maintaining normal cellular structure. In cardiomyocytes, the cytoskeleton plays a vital role in sustaining both contractile and diastolic functions; proper polarity is essential for coordinating electrical signalling between cardiomyocytes and ensuring synchronized contractions. Furthermore, there are interactions among these components, as well as regulatory mechanisms governing them. An imbalance in these interactions might contribute to the pathological processes underlying CMs (Grego-Bessa et al., 2023; Burbaum et al., 2021). In this study, we assessed the expression levels of skeleton and polarity proteins in *Arhgef18* cKO mice, both in the heart tissue and in cardiomyocytes. The results indicated that cytoskeletal proteins (vinculin, αβ-tubulin, cTNT and α-actinin) and polarity proteins (PARD3, SCRIB and CRB2) were downregulated in *Arhgef18* cKO mice compared with in controls. These findings confirm that cardiomyocyte-specific *Arhgef18* cKO results in the aberrant expression of cytoskeletal and polarity proteins. Numerous studies have reported a significant association between the cytoskeleton and CMs. Investigations have identified several regulatory pathways linked to the cytoskeleton and cell polarity, including, but not limited to, the VEGF pathway, PCP pathway and Rho pathway (Tian et al., 2015; Ichida et al., 2001; Theis et al., 2006). In our previous study, we screened the Rho GTPase cycle pathway, which could be a potential signalling pathway of *Arhgef18* in the cytoskeleton and polarity (Guo et al., 2021). We will follow up with RNA sequencing on the mouse heart to look for a related pathway, which will also be the next target signalling pathway to study.

Our study results are similar to those from our previous research (Wan et al., 2023), with both *Arhgef18^flox/flox^*; *Nkx2.5-Cre* mice and *Arhgef18^flox/flox^*; *Myh6-Cre* mice showing abnormalities in cytoskeletal and polarity proteins, confirming that the reduction in these proteins occurs later than the critical period of trabecular development. *Arhgef18^flox/flox^*; Nkx2.5-Cre mice exhibit a CM phenotype, whereas *Arhgef18^flox/flox^*; *Myh6-Cre* mice do not show any related CM phenotypes. This indicates that *Arhgef18* likely plays a crucial role in myocardial development during the 7.5-8.5 days of embryonic development, further confirming that *ARHGEF18* is a key gene in the occurrence and development of CMs.

In conclusion, we successfully established an *Arhgef18* cKO mouse model, which exhibited the clinical phenotype characteristic of CMs. Our findings confirmed cytoskeletal and polarity abnormalities in *Arhgef18* cKO mice. This study provides a new animal model for investigating the pathogenesis of CMs, and carried out in-depth studies on cytoskeletal and cell polarity, providing basic research evidence for the diagnosis, treatment and prevention of CMs.

## MATERIALS AND METHODS

### Mice

*Arhgef18^flox/+^* mice were purchased from Cyagen Biosciences, Inc. As shown in Fig. S2A, a LoxP site was inserted both upstream of exon 3 and downstream of exon 4. *Nkx2.5-Cre* mice were purchased from Shanghai Model Organisms Center, Inc. The *Nkx2.5-Cre* mice, which utilize the *Nkx2.5* promoter to drive Cre recombinase expression for LoxP site-specific recombination, were employed to achieve cardiomyocyte-specific knockout of *Arhgef18* (Zhang et al., 2018; Boczonadi et al., 2014; Courtney et al., 2021; Moses et al., 2001; Kim et al., 2018; Stanley et al., 2002). As shown in Fig. S2B, *Arhgef18^flox/flox^; Nkx2.5-Cre* (*Arhgef18^fl/fl^* cKO), *Arhgef18^flox/+^; Nkx2.5-Cre* (*Arhgef18^fl/+^* cKO), *Arhgef18^flox/flox^* (*Arhgef18^fl/fl^*) and *Arhgef18^flox/+^* mice were obtained after two rounds of crossbreeding with *Arhgef18^flox/+^* and *Nkx2.5-Cre* mice.

In this study, all mice were housed at The Animal Centre for the Children's Hospital of Chongqing Medical University, Chongqing, China, under the environmental regulations of a 12-h light/dark cycle with free access to food and water. The mice were specific pathogen-free C57BL/6 mice. Male and female mice were randomly divided into different experimental groups. All experiments were performed by operators unaware of genotype. No mice were excluded from the analysis. The body weights of the mice were measured at 1, 2, 3, 4, 8 and 12 weeks of age, and the times of births and natural deaths were recorded. The mice were sacrificed via cervical dislocation at 4 weeks of age to obtain heart, liver, lung and kidney tissues. The experimental protocol was approved by the Experimental Animal Management Committee, Children's Hospital of Chongqing Medical University, China (licence number: CHCMU-IACUC2021 1028001).

### Mouse genotyping

Genomic DNA (gDNA) was isolated from mouse toe tissue using a Mouse Direct PCR Kit (Bimake). gDNA amplification was performed with a PCR assay employing 2× M-PCR OPTI™ Mix (Bimake) and gene primers. The DNA was separated by 2% agarose gel electrophoresis, exposed and analysed on an iBright gel imager (Invitrogen). Among the *Arhgef18-Loxp* genotypes, *Arhgef18^fl/fl^* was characterized by a band of 432 bp, *Arhgef18^flox/+^* by bands of 432 bp and 375 bp, and wild type by a band of 375 bp. The *Nkx2.5-Cre* genotype was characterized by a band of 603 bp, and the wild-type genotype was characterized by no band. The primer sequences are provided in Table S2.

### q-PCR

Total RNA was isolated from heart, liver, lung and kidney tissues using a SimplyP Total RNA Extraction Kit (BioFlux). Single-strand cDNA synthesis was performed using an Evo M-MLV RT Mix Kit with gDNA Clean (Accurate Biology). cDNA amplification was performed according to a quantitative real-time PCR assay employing a SYBR Green premix Pro Taq HS qPCR Kit (Accurate Biology). *18S* (also known as *Rn18s*) was used as the housekeeping gene to normalize the transcription levels of the genes of interest. Relative gene expression was calculated via the 2^−ΔΔCt^ method. The primer sequences are provided in Table S3. Four to six mice heart specimens per group were used.

### Western blotting

Total protein was isolated from heart, liver, lung and kidney tissues via an ice-cold mixture of RIPA buffer, protease inhibitor and phosphatase inhibitor (Beyotime). The protein concentration was measured using a BCA kit (Beyotime). The proteins were separated by SDS‒PAGE and then transferred to polyvinylidene fluoride membranes (Millipore). The membranes were blocked for 1 h with 5% milk and then incubated overnight with primary antibody at 4°C, followed by 2 h with secondary antibody at room temperature. The membranes were exposed to enhanced chemiluminescence reagent and visualized with a gel imaging analyser developer (Bio-Rad). HSP90 was used for normalization. Quantitative analysis was performed with Image Lab software (Bio-Rad). Antibody information is provided in Table S4. Four to six mice heart specimens per group were used.

### Echocardiography

Echocardiography was performed with a Vevo 3100 system (FUJIFILM). B-mode and M-mode ultrasound images of paraspinal short-axis sections of the mice were acquired to measure relevant parameters. The mice were anaesthetized with isoflurane (3-4 l/min induction, 0.5-1 l/min maintenance), and their body temperature (36-37°C) and heart rate (480-520 beats/min) were maintained during echocardiographic inspection. The measurements were recorded as the average of at least three consecutive cardiac cycles. The LVEF, LVFS, LVAW, LVID and left-ventricular volume were analysed with Vevo LAB 5.8.0. Five to eight mice per group were used.

### H&E staining

The mice were sacrificed via the cervical dislocation method, and the whole heart was removed and washed in PBS. After fixation in 4% paraformaldehyde for 48 h, gradient dehydration, paraffin embedding, 5 μm continuous heart longitudinal sectioning, deparaffinization, hydration, Haematoxylin staining, Eosin staining, dehydration, transparency, sealing and air drying were performed. The images were captured and analysed on a microscanning instrument (Shengqiang Full Slide Scanner, Shengqiang Technology in China). Three mice per group were used.

### Immunofluorescence

Cardiomyocytes were obtained from 4-week-old mice via the Langendorff perfusion system (Chengdu Instrument Factory). Cardiomyocytes were fixed with 4% paraformaldehyde for 15 min in the dark, permeabilized with 0.1% Triton X-100 for 10 min, and blocked with 5% bovine serum albumin goat serum blocking solution for 1 h at room temperature. The cells were incubated with primary antibodies overnight at 4°C, incubated with secondary antibodies at room temperature in the dark for 1 h, and stained with 4′,6-diamidino-2-phenylindole (DAPI) in the dark for 10 min. Cardiomyocyte images were acquired and statistically analysed under a fluorescence inverted microscope (Nikon) using a glass-bottomed cell culture dish (Biosharp). Antibody information is provided in Table S4. Three mice per group were used, with >20 cardiomyocytes per mouse.

### Statistical analysis

All data were analysed using Prism 8.3.0 (GraphPad Software). Normally distributed continuous variables are presented as the mean±s.d. and were compared via one-way ANOVA followed by Tukey's, Dunnett's or Dunn's multiple comparison post hoc tests. The survival rates of the mice were analysed via the log-rank test. *P-*values less than 0.05 were considered statistically significant.

## Supplementary Material

10.1242/dmm.052172_sup1 Supplementary information
