# Supplementary material for: Establishment and functional studies of a model of cardiomyopathy with cardiomyocyte-specific conditional knockout of Arhgef18
Source: Dis Model Mech. 2025 Mar 31;18(3):dmm052172. doi: 10.1242/dmm.052172 (PMC11992352; doi:10.1242/dmm.052172)
Supplement: Supplementary information [file dmm-18-052172-s1.pdf]

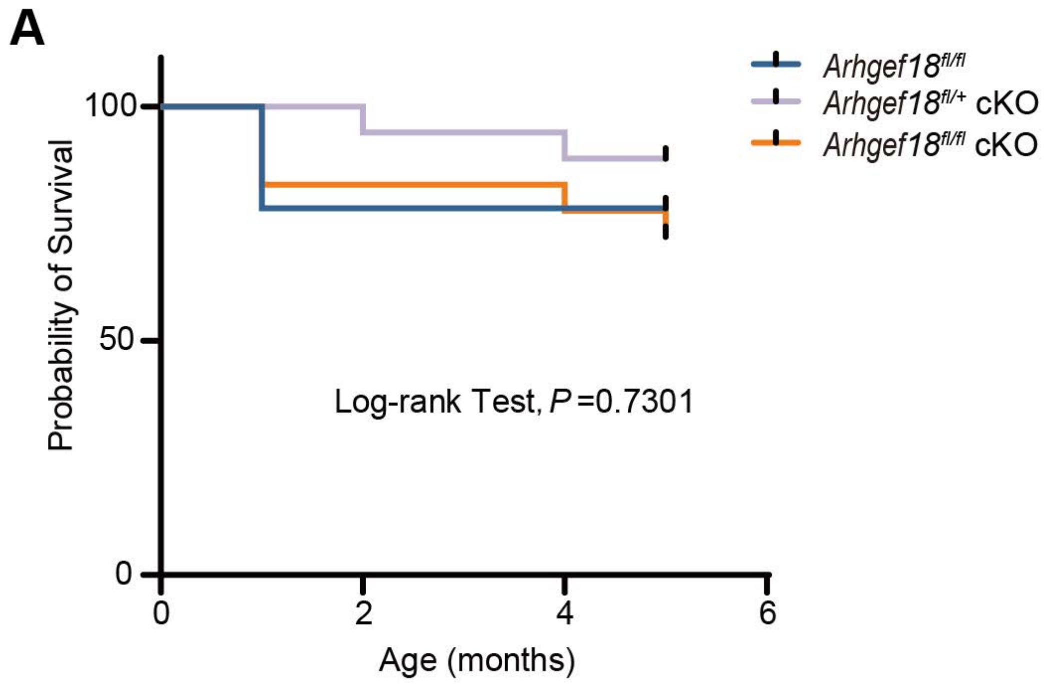

Fig. S1. Kaplan–Meier survival curves. (A) With no statistically significant difference in survival curves among *Arhgef18*$^{fl/fl}$ (n=23), *Arhgef18*$^{fl/+}$ cKO (n=18), and *Arhgef18*$^{fl/fl}$ cKO (*n*=18) mice according to the log-rank test.

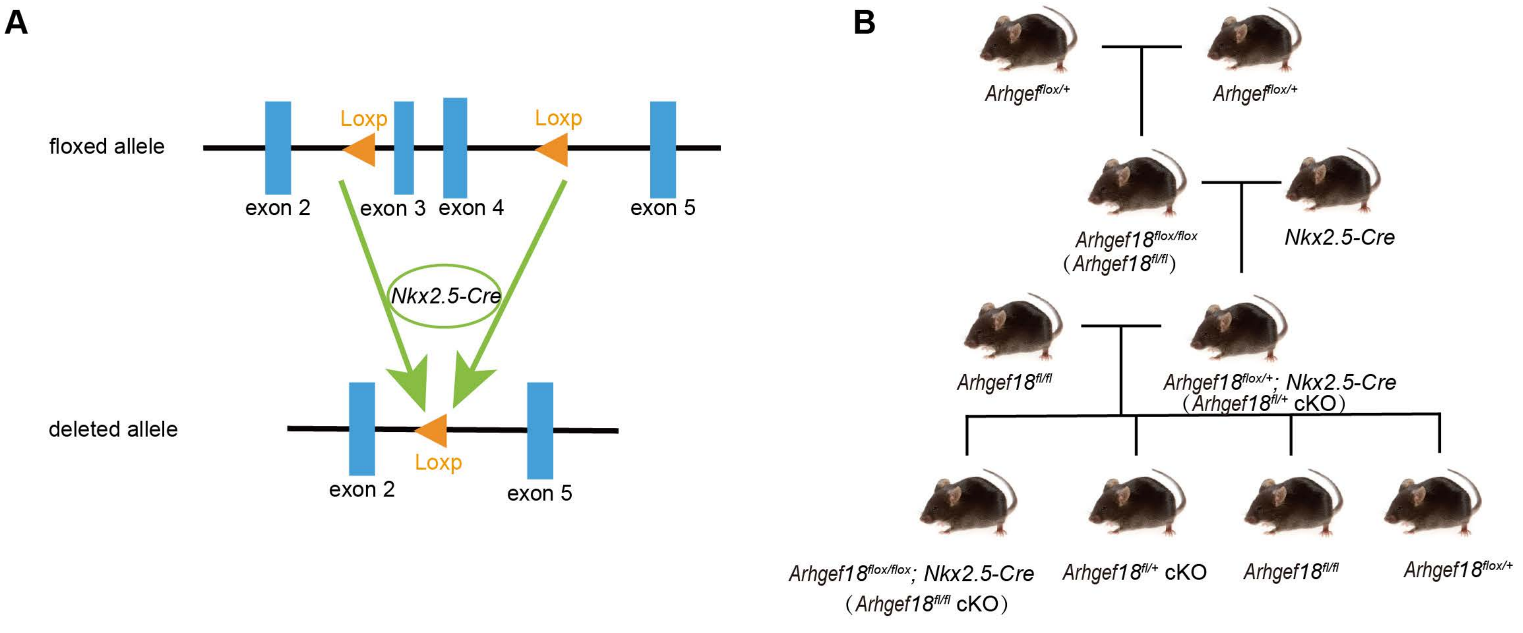

Fig. S2. Construction and breeding of *Arhgef18* cKO mice. Diagram of the construction strategy for *arhGEF18* cKO mice. (B) Flowchart for breeding *Arhgef18* cKO mice.

Disease Models & Mechanisms • Supplementary information

Table S1. Cardiac function measurements using echocardiography.

Parameters	2 W			4 W			12 W			24 W		
	Arhgef18$^{fl/fl}$	*Arhgef18*$^{fl/+}$ cKO	*Arhgef18*$^{fl/fl}$ cKO	*Arhgef18*$^{fl/fl}$	*Arhgef18*$^{fl/+}$ cKO	*Arhgef18*$^{fl/fl}$ cKO	*Arhgef18*$^{fl/fl}$	*Arhgef18*$^{fl/+}$ cKO	*Arhgef18*$^{fl/fl}$ cKO	*Arhgef18*$^{fl/fl}$	*Arhgef18*$^{fl/+}$ cKO	*Arhgef18*$^{fl/fl}$ cKO
**Heart rate (bpm)**	493.7±15.19	504.1±8.99	501.4±9.24	498.2±16.96	515±12.26	498.6±13.93	507.2±5.30	515.5±5.21	508.8±3.38	508.1±13.48	497.1±14.64	504.8±11.76
**LV end-diastolic**												
**LVAW (mm)**	0.75±0.15	0.80±0.11	0.70±0.19	0.85±0.11	0.77±0.17	0.68±0.14	1.09±0.15	1.04±0.34	0.81±0.12	1.08±0.10	1.08±0.13	0.92±0.17
**LVID (mm)**	2.44±0.33	2.19±0.12	2.35±0.39	3.05±0.41	3.17±0.23	3.11±0.24	3.48±0.46	3.52±0.09	3.80±0.54	3.21±0.44	3.25±0.27	3.74±0.50
**LVPW (mm)**	0.67±0.11	0.67±0.16	0.60±0.11	0.68±0.14	0.69±0.05	0.66±0.04	0.78±0.11	0.76±0.20	0.77±0.04	0.94±0.15	0.95±0.28	0.86±0.20
**LV volume (μl)**	21.57±7.00	16.13±2.23	20.01±8.84	37.33±11.52	40.43±6.81	38.68±6.99	51.24±16.89	51.56±3.39	63.62±20.28	51.24±14.20	51.56±8.14	63.62±20.37
**LV end-systolic**												
**LVAW (mm)**	1.25±0.22	1.27±0.11	1.17±0.17	1.41±0.15	1.32±0.21	1.09±0.24*	1.80±0.25	1.71±0.30	1.38±0.10*	1.62±0.19	1.56±0.14	1.30±0.26*
**LVID (mm)**	1.11±0.26	1.09±0.11	1.18±0.36	1.65±0.23	1.80±0.14	1.97±0.15**	1.84±0.41	1.97±0.18	2.49±0.44*	2.02±0.33	2.16±0.33	2.94±0.61**†
**LVPW (mm)**	1.16±0.12	1.10±0.16	0.98±0.17	1.08±0.15	1.14±0.11	1.05±0.10	1.39±0.19	1.26±0.26	1.15±0.09	1.34±0.19	1.28±0.25	1.09±0.15
**LV volume (μl)**	3.02±1.69	2.64±0.67	3.84±3.56	8.00±2.69	9.84±1.91	12.42±2.32*	11.13±6.82	12.38±2.83	23.07±10.18*	13.62±5.24	16.04±5.84	35.27±19.46*†
**LVEF (%)**	86.86±4.77	83.73±3.00	83.3±6.63	78.65±2.22	75.73±1.48	67.54±4.91***††	79.52±5.46	76.06±4.81	64.31±6.94**†	67.57±9.41	63.33±8.74	44.62±11.08**†
**LVFS (%)**	54.97±5.89	50.45±3.88	50.58±6.54	45.95±2.25	43.26±1.28	36.51±4.01***††	47.53±5.19	44.1±4.62	34.67±4.95**†	37.02±7.58	33.74±6.50	21.87±6.04**†
**LV mass (mg)**	46.85±18.94	40.85±8.97	39.62±18.79	72.27±20.76	72.81±19.64	61.25±12.51	118.7±29.04	112.6±11.80	106.7±25.73	118.3±36.37	119.4±29.85	122.8±25.35
**LV mass Corrected (mg)**	37.48±15.15	32.68±7.18	31.69±15.03	57.82±16.61	58.25±15.71	49.00±10.01	94.95±13.23	90.11±9.44	85.36±20.59	94.67±29.10	95.55±23.88	98.26±20.28

Values are expressed as mean ± s.d. from at least 5-8 mice per group. Statistical significance was determined by ANOVA.

$^{*}P<0.05$ $^{**}P<0.01$ $^{***}P<0.001$ compared to *Arhgef18*$^{fl/fl}$, $^{\dagger}P<0.05$ $^{\dagger\dagger}P<0.01$ $^{\dagger\dagger\dagger}P<0.001$ compared to *Arhgef18*$^{fl/+}$ *cKO*.

Table S2. Primer sequences of PCR.

Gene	Forward primer (5'-3')	Reverse primer (5'-3')
Arhgef18-Loxp	GCTTCAGACTCACATAGCACTGAG	TAGCTAGGTAGCACAAGCCTCA
Nkx2.5-Cre	GCCTCCGCCAACAGCAA	CCCAGATCAGATCCCATACAATG

Disease Models & Mechanisms • Supplementary information

Table S3. Primer sequences of q-PCR.

Gene	Forward primer (5'-3')	Reverse primer (5'-3')
Arhgef18	TCTTAGTCTCCAGGCGGTCA	CTCCTCACGCTGCTTCTCAA
vinculin	CATGGTGATGGATGGCCAAGG	GTTCAAGGTCTGGGTGGAGGA
αβ-tubulin	TGTTCGTAGACCTGGAACCC	CCTTGCCAATGGTGTAGTGGG
α-actinin	GGGTTACTCCTGTGGTGTCA	CTCTCTGCCACATCAAAGGC
cTNT	CGACCACCTGAATGAAGACCA	TCCCAGGAGTTTTGGAGACTT
Pard3	TCTACTGGGCAAAGCCAACC	ATCCTGCGTTCTCGGTCATC
Scrib	GCGAGTGTGGAGGGAAAGAA	ATGACGCTGACGGAGGGATA
18S	CGCCGCTAGAGGTGAAATTCT	CGCCGCTAGAGGTGAAATTCT
Nppa	GCTTCCAGGCCATATTGGAG	GGGGGCATGACCTCATCTT
Nppb	GAGGTCACTCCTATCCTCTGG	GCCATTTCCTCCGACTTTTCTC

Table S4. Antibodies used for western blots and immunofluorescence.

Protein	Product Number	Company	Country	WB	IF
Arhgef18	GTX102223	GenTex	USA	1:500	-
vinculin	v9131	Sigma	Germany	1:1000	1:800
αβ-tubulin	2148	Cell signaling	USA	1:1000	1:50
α-actinin	6487	Cell signaling	USA	1:1000	1:200
cTNT	ab209813	Abcam	USA	1:1000	1:1000
SCRIB	27083-1-AP	Proteintech	China	1:1000	-
CRB2	ab156286	Abcam	USA	1:1000	-
hSP90	TA500494	OriGene	China	1:2000	-
Goat anti-rabbit IgG (H+L)	SA00013-2	Proteintech	China	1:5000	-
Goat anti-mouse IgG (H+L)	SA00001-1	Proteintech	China	1:5000	-
555 HRP-goat anti-rabbit IgG	Y6088L	UELandy	China	-	1:200
488 HRP-goat anti-mouse IgG	Y6104L	UELandy	China	-	1:200
